# Wireless E-Nose Sensors to Detect Volatile Organic Gases through Multivariate Analysis

**DOI:** 10.3390/mi11060597

**Published:** 2020-06-18

**Authors:** Saifur Rahman, Abdullah S. Alwadie, Muhammed Irfan, Rabia Nawaz, Mohsin Raza, Ehtasham Javed, Muhammad Awais

**Affiliations:** 1 Electrical Engineering Department, College of Engineering, Najran University, Najran 61441, Saudi Arabia; asalwadie@nu.edu.sa (A.S.A.); miditta@nu.edu.sa (M.I.); 2 Department of Physics, COMSATS University, Park Road, Chak Shahzad Islamabad 45550, Pakistan; rabianawaz4477@gmail.com; 3 Department of Computer and Information Sciences, Northumbria University, Newcastle upon Tyne NE1 8ST, UK; mohsinraza119@gmail.com; 4 Helsinki Institute for Life Sciences, Neuroscience Center, University of Helsinki, 00014 Helsinki, Finland; ehtasham.javed@helsinki.fi; 5 Energy and Environment Institute, Faculty of Science and Engineering, University of Hull, Hull 7RX, UK

**Keywords:** gas sensors, principal components analysis, multivariate analysis, metal oxide semiconductor (MOS) sensors, electronic, detection, electronic nose

## Abstract

Gas sensors are critical components when adhering to health safety and environmental policies in various manufacturing industries, such as the petroleum and oil industry; scent and makeup production; food and beverage manufacturing; chemical engineering; pollution monitoring. In recent times, gas sensors have been introduced to medical diagnostics, bioprocesses, and plant disease diagnosis processes. There could be an adverse impact on human health due to the mixture of various gases (e.g., acetone (A), ethanol (E), propane (P)) that vent out from industrial areas. Therefore, it is important to accurately detect and differentiate such gases. Towards this goal, this paper presents a novel electronic nose (e-nose) detection method to classify various explosive gases. To detect explosive gases, metal oxide semiconductor (MOS) sensors are used as reliable tools to detect such volatile gases. The data received from MOS sensors are processed through a multivariate analysis technique to classify different categories of gases. Multivariate analysis was done using three variants—differential, relative, and fractional analyses—in principal components analysis (PCA). The MOS sensors also have three different designs: loading design, notch design, and Bi design. The proposed MOS sensor-based e-nose accurately detects and classifies three different gases, which indicates the reliability and practicality of the developed system. The developed system enables discrimination of these gases from the mixture. Based on the results from the proposed system, authorities can take preventive measures to deal with these gases to avoid their potential adverse impacts on employee health.

## 1. Introduction

In present times, industries have seen the importance of sensory olfactory systems, in that they can help in detecting the adverse effects of gases on human health. An electronic nose (e-nose) refers to a system that mimics the olfactory system of humans with regards to its functions in order to complete qualitative and quantitative analyses of gases or odors, which is also known as the simulated system of olfaction [1]. The e-nose that functions as a machine/computer has been applied in many areas. An e-nose follows appropriate signal conditioning and processing to produce a unique pattern that helps discriminate different types of odors. The e-nose is a smart intelligent system that is comprised of sensors that respond to smell, as well as convert chemical information into electrical signals.

Moreover, these sensors can be integrated into an e-nose using advanced micro/nano-fabrication technologies. There are several applications based on e-nose, such as the e-nose being used in defense divisions and pharmaceutical laboratories, in areas such as food processing industries, cosmetics industries, environmental condition detection and diverse scientific fields of research [2]. As the sensor is exposed to odor/gas, its resistance changes accordingly [3]. The different classification methods like principal component analysis (PCA) and partial least squares (PLS) or gas chromatography (GC) can help detect gas samples according to the odor and concentration of different gases. Different classification methods are used to detect and differentiate various gases from mixtures. Different classification methods are related to different multivariate analysis methods, such as PCA, Gaussian mixture models (GMMs), neural networks, and so forth [3]. A sample’s odor can be compared using sensors arrays. This causes a minor change to the chemical or physical properties of a material, which as a result, introduces a readable change in the electrical properties (e.g., conductivity) of the material [4]. The e-nose reduced the detection period from 65% to 30% (time) by detecting compounds within 16 h instead of taking 1–2 days [5].

Fan et al. [6] have described the supervised learning techniques used for e-nose data analysis and classification, which requires several datasets to impose a limitation for using these methods because a large number of datasets for organic gases—i.e., A, E and P—are unavailable. Instead, in a free environment, a gas is classified via unsupervised learning. Unsupervised learning has more significant advantages over supervised learning because the classification of different gases uses an e-nose. Monroy et al. [7] carried out an experimental analysis of ethanol and acetone on a moving platform rather than a stable platform. In another study, an e-nose was used to experimentally classify compounds such as limonene, ethanol, and dimethyl sulfide [8]. In the gas detection area, a comprehensive review was conducted by Hodgkinson and Tatam [9], which compared methods like photoacoustic spectroscopy, spectrophotometry, tunable diode laser spectroscopy, and non-dispersive infrared. However, they did not consider any multivariate techniques, such as PCA, to detect and classify a mixture of gases. In another study [10], a system to detect and classify carbon monoxide and oxygen was developed. However, a mixture of A, E and P was not considered for the classification [10]. Several techniques were again reviewed by Rydosz et al. [11] for the detection of acetone to analyze the health condition of diabetic patients. However, the combination of organic gases A, E and P was not considered. There are several customized devices that can detect one gas at a time (such as ethanol and acetone gases), yet they are unable to differentiate more than one gas from a mixture [12]. Moreover, instrumental methods to determine volatiles such as gas chromatography-mass spectrometry (GC-MS) are expensive and require trained personnel [13]. Thus, the study conducted and presented in this paper aimed to differentiate and classify the mixture of three volatile gases—i.e., A, E and P—using PCA. The proposed technique classified gases by making boundary clusters for the three gases A, E and P. At present, the e-nose has been widely studied and applied in medical diagnostics [14], food quality testing [15], and environmental monitoring [6].

Table A1 (given in Appendix B) indicates different compounds analyzed by different methods and provides the limitations associated with each. At present, research conducted by Fan et al. [6] has only considered the differentiation of A, E, and P from mixture through the Knuth–Morris–Pratt (KMP) algorithm. However, the KMP algorithm has a prominent limitation, i.e., the poor separability of gas mixtures. These gases are mostly produced in oil and petroleum refineries and can cause different diseases in human beings [6]. Acetone can cause unconsciousness and possibly coma, ethanol can cause heart diseases, and propane can cause suffocation and irregular heartbeat. To avoid these diseases, the detection and differentiation of gases from the mixture is necessary so that people working in oil and petroleum refineries can avoid exposure to these gases in a timely measure. Metal oxide semiconductor (MOS) electronic sensors play an important role in sensing such gases and can provide an alarm to prevent themselves and others from the potential exposure to such gases. Thus, this study further aims to develop a novel MOS-nose sensor-based detection system to classify such volatile gases from a mixture of gases and further classify them using PCA based on a multivariate analysis technique. The contributions of the proposed work are:

- To probe and differentiate commonly produced gases from mixtures (acetone, ethanol, and propane) in manufacturing industries using a high performance PCA-based multivariate analysis technique.
- To identify various clusters of these (acetone, ethanol, and propane) using a PCA numerical test.

### Metal Oxide Semiconductors (MOS) Gas Sensors

MOS gas sensors can sense a significant amount of hazardous gases. The electrical response of these sensors to the gas concentration is expressed as: R = log a (C), where R is the electrical response and C is the concentration. MOS gas sensors show poor selectivity and their electrical responses to different gases vary over a large magnitude span. The sensors are used in an array of 4–32 sensors and all have different selectivities that can attain a unique, complete sample or fingerprint that resembles the overlying reaction of the different sensors to the range of compounds within the sample. To improve selectivity, the use of such arrays leads to a convincing amount of redundant information, which is very useful if one of the sensors fail.

The sole advantage of an MOS gas sensor is that it can operate at room temperature. However, its response time is too long (20–40 s) and cannot be used for fast analysis. Moreover, this sensor is not robust, and its performance is affected by temperature fluctuation and aging [16]. Some sensors have limitations in the food industry because of their high compassion to compounds such as ethanol, CO_2_, or humidity [17].

Different types of MOS gas sensor arrays such as TGS 2600, TGS 2602, TGS 2611, and TGS 2620 are used in this work (see: Appendix C). All of these sensors consist of features such as high sensitivity to volatile organic compounds (VOCs) and odorous gases, low power consumption, portable in size, high sensitivity to gaseous air, contaminants, long life, low cost, and simple electrical circuits.

## 2. The Methodology of Proposed MOS Sensor-Based E-Nose Detector

**Figure 1:**
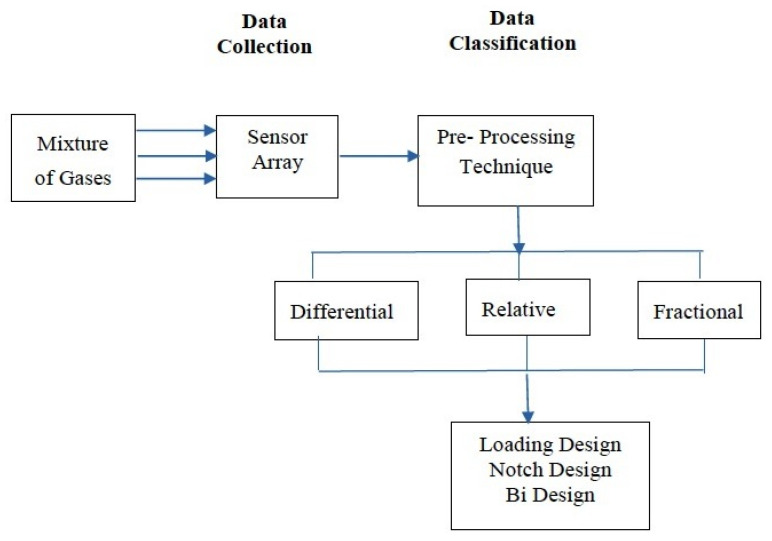
Block diagram of gases—data collection and classification.

The block diagram for the flow of gas sensing and classification is shown in Figure 1. The MOS sensor (sensor array) collects the gas mixture produced by an industrial plant, and then, further processes the mixture via a preprocessing technique using PCA. 

**Figure 2:**
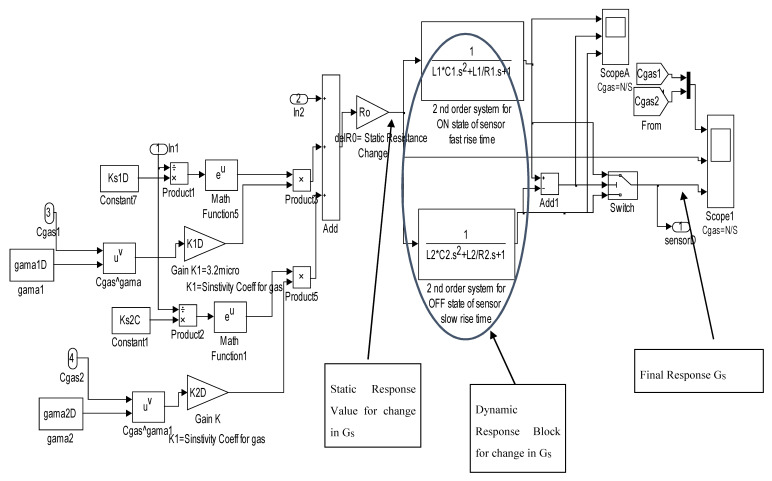
Simulink model of classifying a mixture of gases for each sensor.

Based on the block diagram proposed in Figure 1, an initial simulation was carried out on the gas mixture. Figure 2 shows the Simulink model of differentiating a gas mixture using sensors with specific parameters according to different gases.

This preprocessing step uses three types of analysis: differential, relative, and fractional. These three types have loading design, notch design, and Bi design plots that are used to classify gases. The loading design demonstrates the performance of the sensor array used for differentiating the variations of sensor measurements. It also shows the correlation between the set of sensors. Notch design examines data differences. It also discriminates against the mixture by forming a cluster boundary around the gases. The Bi design shows the loading and notch design in one plot. In Bi design, both variables and observations are made together.

## 3. Theory of Principal Component Analysis

A multivariate classification can be performed using a technique such as PCA. In this study, preprocessing was conducted via odor sampling. Afterwards, specified data were detached and information was stored in a computer system for further analysis. In data classification, the algorithm was examined and realized. To analyze odors, three technologies were used: differential, relative, and fractional (details are appended in Appendix A). PCA used data dimension reduction and clustering methods [18]. The authors in reference [19] used a deep convolutional neural network (DCNN) to classify gases. The critical part of the data analysis was to consider PCA as an eigenvector test of the data report, featuring a fragment into another coordinate framework. By smothering data elements, the critical part of PCA changed the primary data information. In the original data information, using less factors showed that a different pattern of change existed. Accordingly, as a preprocessing procedure, PCA was used.

To confirm the pattern, PCA was used and the analysis of the limited sample circulation using the electronic compress nose was completed. All related PCA data depend on a statistical strategy, through which all associated data can easily be envisioned [20]. In experiments and logic, the PCA strategy incorporates the following steps:

(i) The matrix X_M×N_ gives the information on row, which is represented by M, demonstrating that different redundancies happen amid the experiment. Column N delineates non-subordinate sensors.
(ii) Normalization and arrangement of data is performed in matrix form Norm (X_M × N_) with a mean reduction. Subtraction and estimation of the average of each N column from the informational data is collected. This new information data quantity makes the mean equivalent to zero.
(iii) Measurement of the covariance matrix is performed as Cov (X_M×N_), which helps discover eigenvectors and eigenvalues of the covariance matrix. The created eigenvectors ought to be unit eigenvectors.
(iv) The eigenvalues and eigenvectors are arranged and adjusted. The eigenvalues are adjusted from eigenvectors from maximum to minimum (Cov (X_M×N_)) with max→min.
(v) The output of PCA is restored by using the product of a matrix, transposed and given as ((Cov (X_M×N_))max→min*Norm (X_M×N_))^T^. Further to this, the accomplished informational data collection with orthogonal linear change presented in 2D/3D also includes free informational data collection.

**Figure 3:**
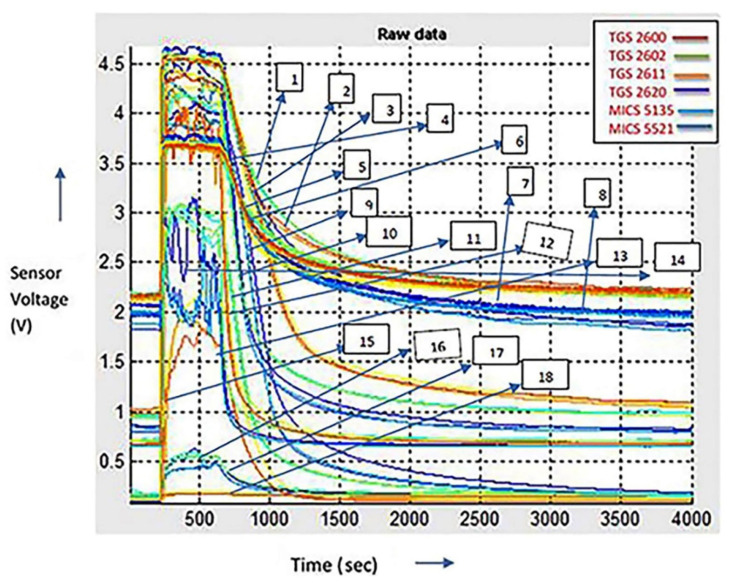
Time response of sensors based on metal oxide semiconductor (MOS) techniques for three various samples of volatile organic compounds (VOCs), each having three statistical records for the database (D).

For the simulation performed in this study, real-time data were taken from the Mobile Robot Programming Toolkit with a high-quality standard. The collected data consisted of MOS sensor voltage feedback with a MATLAB arrangement cluster. This dataset consists of voltage signals from MOS gas sensors. For different types of volatile organic compounds (VOCs), such as A, E, and P, six MOS sensors recorded data for about 4000 s. The real-time response curves are shown in Figure 3. In the figure below, the curves are labelled from point 1 to 18. This figure shows an arbitrary representation to show how different sensors behave differently, as three different gases’ responses are shown in the figure using six sensors. There are 18 curves representing the raw data of the three gases using six sensors. Points 1 to 6 show the curves for gas A. Point 1 shows the response of TGS 2602, point 2 represents sensor TGS 2611, point 3 shows the sensor response of TGS 2600, point 4 shows the sensor TGS 2620, point 5 shows the response of MICS 5135, point 6 shows the sensor response of MICS 5521. In the same manner, points 7 to 12 show the curves for gas E: point 7 shows the response of sensor TGS 2620, point 8 represents the sensor MICS 5135, point 9 depicts the response of MICS 5521, point 10 shows the response of TGS 2602, point 11 shows the sensor response of TGS 2611, point 12 represents the sensor response of TGS 2600. In a similar way, points 13 to 18 show the curves for gas P, point 13 shows the response of TGS 2620, point 14 shows the response of sensor MICS 5521, point 15 shows the response of TGS 2600, point 16 shows the response of the sensor TGS 2602, point 17 shows the response of MICS 5135, point 18 shows the response of TGS 2611. From this figure, we can see that all the six sensors show different responses for different gases.

All MOS sensors (TGS 2600, TGS 2602, TGS 2611, TGS 2620, MICS 5135, and MICS 5521) responded to the mixture of gases, even at a lower concentration level (1–30 ppm), as depicted in Figure 3. The voltage signals extracted from the MOS sensor response were analyzed using three different analysis methods to determine if the mixture concentration was distinguishable or not. The analysis processes performed were load design, notch design, and Bi design plots.

## 4. Result and Discussion

In the section below, we present the results of three preprocessing techniques used to identify and classify three gases using PCA.

### 4.1. Data Difference Preprocessing Used for Principal Component Analysis of Volatile Organic Compounds and Gases Data

**Figure 4:**
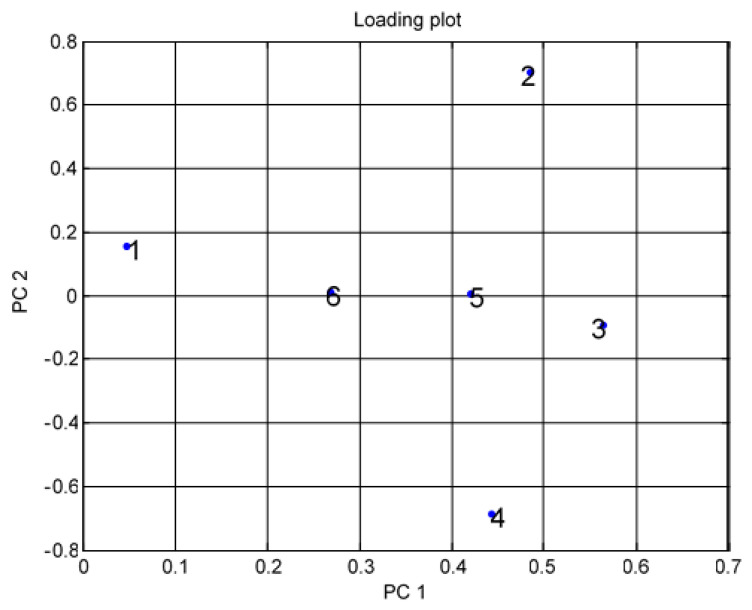
Multivariate technique on organic gases for different feature loading designs.

Preprocessing data for VOCs such as A, E, and P was conducted using PCA. The loading design for the performance of MOS sensor arrays was used for differentiation in the variations of voltage readings (Figure 4). Herein, we present one to six different irregular points in this design. In Figure 4, point one shows TGS 2600, point two shows TGS 2602, point three shows TGS 2611, point four shows TGS 2620, point five shows MICS 5135, and point six shows MICS 5521.

**Figure 5:**
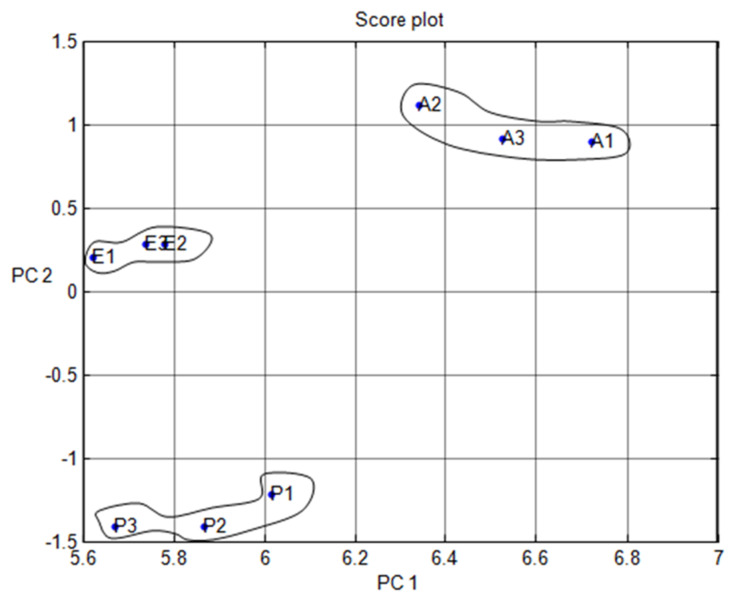
Multivariate technique on organic gases for different feature notch design.

**Figure 6:**
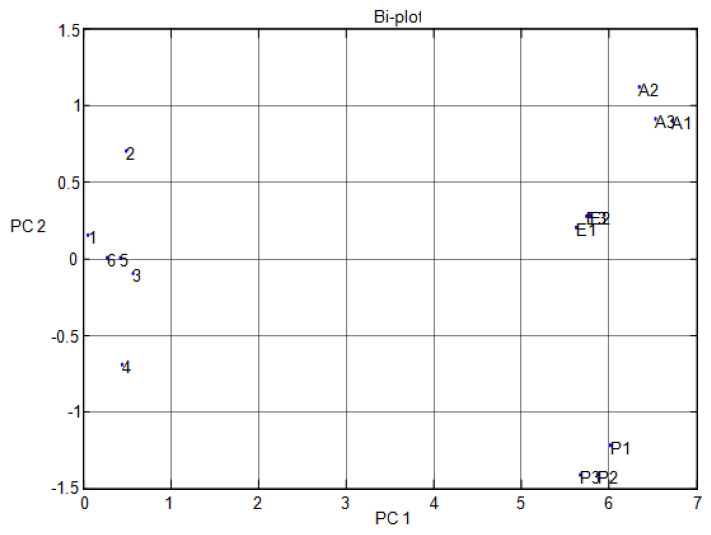
Multivariate technique on organic gases for different features in the Bi design.

Figure 4 shows the results of the data difference design. It had no sensor set response correlated with the other. Figure 5 shows that the notch design for multivariate techniques examines the relationships among data difference considerations. Here, a multivariate method PCA was used to discriminate all three clusters of A, E, and P, represented in one cluster each, using a separate boundary. Figure 5 also shows that PCA can discriminate all these three clusters by the separate boundary. In Figure 6, both variable scatter models and observations are presented together. On the left part of Figure 6, the principal component of MOS sensors is presented, while observations of gas samples are presented on the right side.

### 4.2. Data Fractional Preprocessing Used for Principal Component Analysis of Volatile Organic Compounds and Gas Database

**Figure 7:**
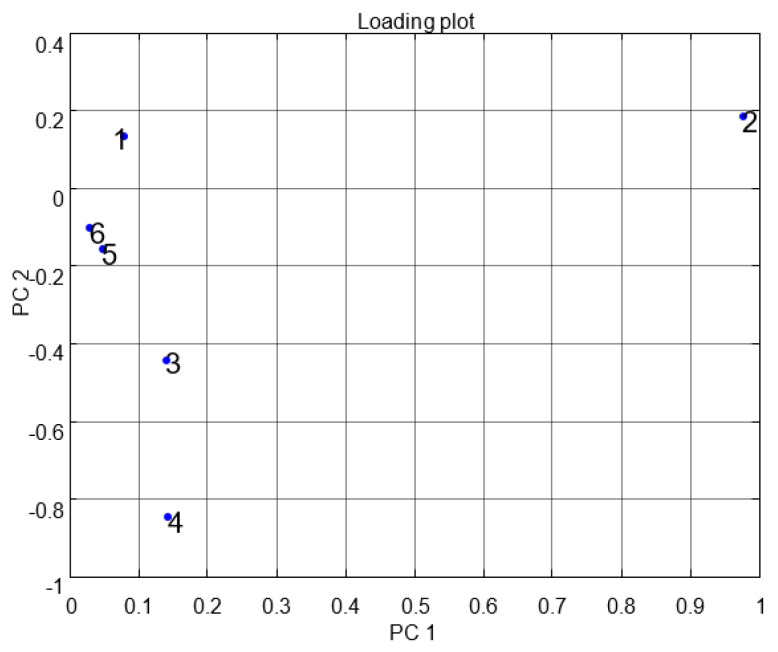
Multivariate technique on organic gases fractional feature loading design.

**Figure 8:**
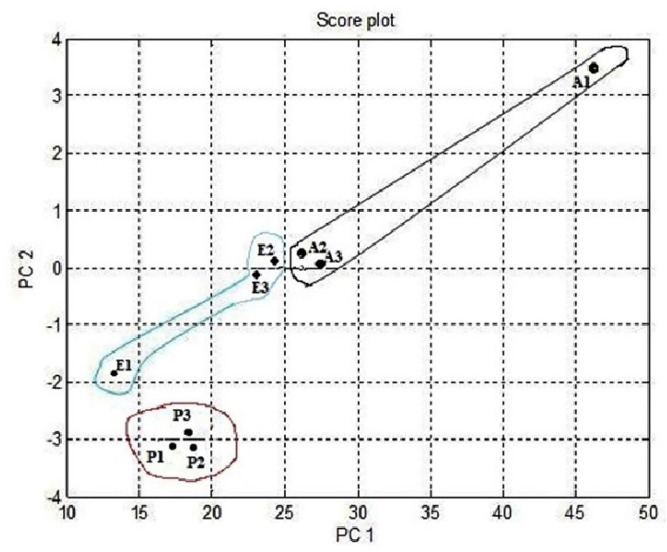
Multivariate technique on organic gases fractional feature notch design.

**Figure 9:**
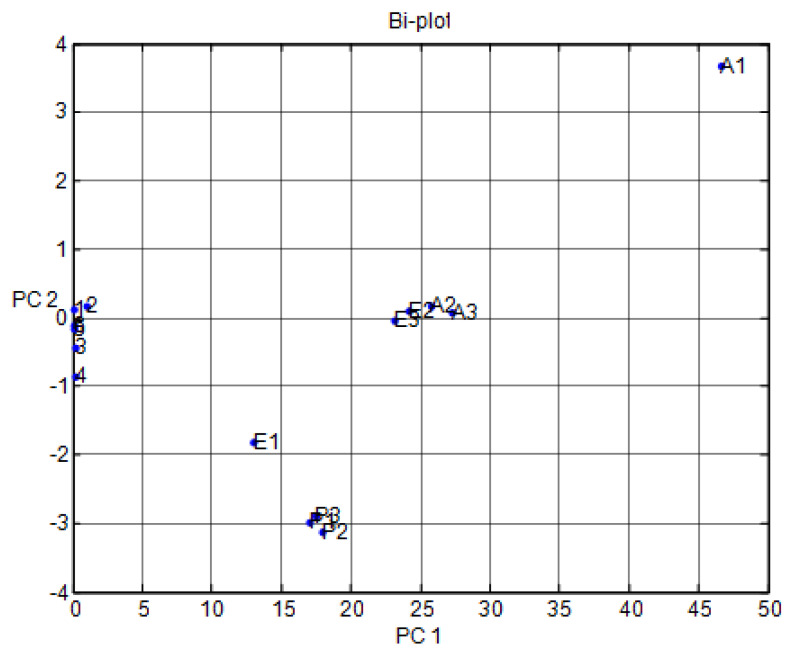
Multivariate technique on organic gases fractional feature Bi design.

Figure 7 indicates the results of the data fractional design. It shows that the MOS sensor set {5,6} responses were correlated with each other or had the same properties. In Figure 7, point one shows TGS 2600, point two shows TGS 2602, point three shows TGS 2611, point four shows TGS 2620, point five shows MICS 5135, and point six shows MICS 5521. The responses were opposite to each other for the other sets of MOS sensors. The notch design shown in Figure 8 inspects the coordination among the study of data difference. Figure 8 further shows that the multivariate technique (PCA) can discriminate against all three clusters of A, E, and P by the separate boundary. Hence, PCA helps classification in incomplete data preprocessing methods, which can ultimately help differentiate the three gases from the mixture. Figure 9 summarizes the results of the loading and notch designs, known together as the Bi design. Both variable scatter models and observations are shown in Figure 9. On the left part of Figure 9, the principal MOS sensor components are presented, while the observation of gas samples are presented on the right side of Figure 9.

### 4.3. Data Relative Preprocessing Used for Principal Component Analysis of Volatile Organic Compounds and Gas Database

**Figure 10:**
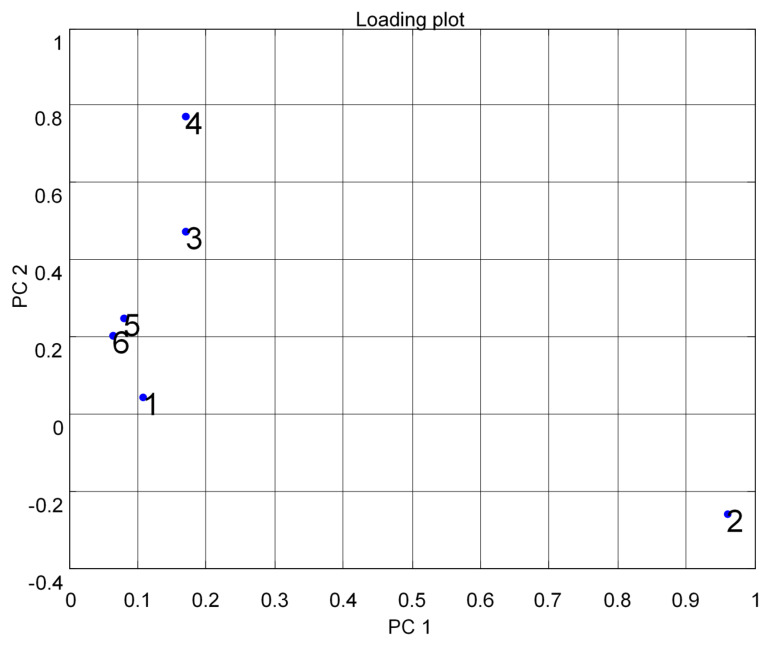
Multivariate technique on organic gases relative feature loading design.

Figure 10 shows the results of the relative data design. In Figure 10, point one shows TGS 2600, point two shows TGS 2602, point three shows TGS 2611, point four shows TGS 2620, point five shows MICS 5135, and point six shows MICS 5521. It shows that the MOS sensor set {5,6} responses were correlated with each other or had the same properties. MOS sensor 2 had a response different from other sensors and groups of sensors {2,3,4} play an important role in classification. 

**Figure 11:**
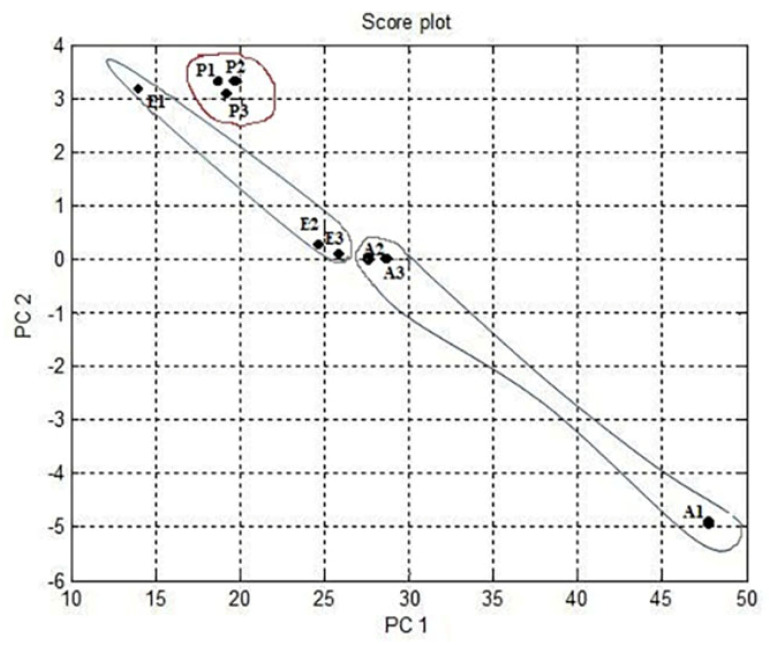
Multivariate technique on organic gases relative feature notch design.

**Figure 12:**
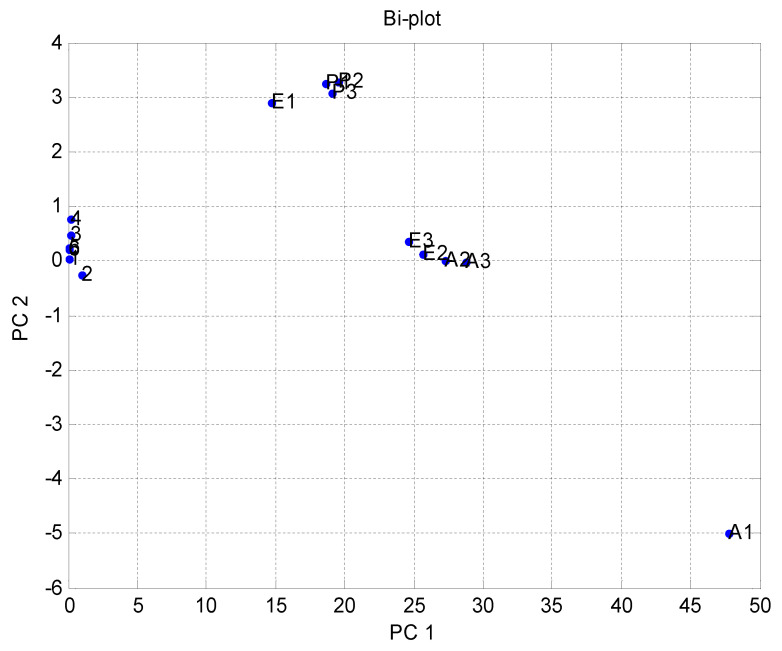
Multivariate technique on organic gases relative feature Bi design.

The notch design shown in Figure 11 investigated the relationships between the study of data differences. Figure 11 also shows that the multivariate technique (PCA) can discriminate against all three clusters of A, E, and P by the separate boundary. Hence, PCA is helpful in the relative data preprocessing method to classify all three gas samples. Figure 12 summarizes the results of the loading and notch design together in one design known as the Bi design. Both variable scatter models and observations are shown in Figure 12. As can be seen, the principal sensor components are presented on the left part of Figure 12 while observation of gas samples are seen on the right part of Figure 12. The primary sensor component was usually distributed and did not show the same act for preprocessing done by relevant data. The information’s point was too crowded because it became difficult to distinguish the smell by the relative method.

According to the different concentration of gases, various sensors are evaluated in Table 1. For the dataset, the PCA technique used for sensors {5} and {6} shows similar responses to gases regarding the fraction and relative preprocessing method. However, the opposite is true regarding the difference preprocessing technique, where none of the sensors have an interaction. The opposite performance is always demonstrated by sensors {3} and {4}. Sensors {2}, {3}, and {4} play the utmost important role. PCA has quite similar results in categorizing the sensors present in this database.

Table 2 shows a comparison between this paper’s proposed method with past studies. Prior research has implemented MIR-PLS, GC-MS, and PLS techniques for differentiation and classification of A, E, and P. Each study presented in Table 2 used either A, E, or P, but did not consider the mixture of these three gases. Additionally, the scope of their studies was focused on detecting human diseases by the gases coming out from petrochemical storages. The proposed method presented in this paper overcame the disadvantage of previous studies by designing multivariate techniques to differentiate and classify the A, E, and P mixture. The three preprocessing methods used here easily organize the gas mixture by making a cluster boundary between the gases. This classification will enable people working in the industry to receive alerts regarding the presence of these gases in the environment and to take preventative measures.

All three pre-handling procedures show them working perfectly without overlapping for each technique, which is also shown in Table 3. Here, the table summarizes classification and overlapping of gases. As depicted in Table 3, each gas sample has different chemical properties, but the preprocessing method works perfectly during the classification of the sample if the sample contains different material properties. Table 3 also shows that there is no overlap of gases found when using the three preprocessing methods.

## 5. Conclusions

The e-nose developed using various MOS sensors has become a widespread practice in different fields. In this study, an e-nose sensor-based volatile gas detection system was developed through multivariate analysis. The proposed system assessed organic gas quality, where a metal oxide sensor was used to collect the data. This was then further analyzed using PCA-based multivariate classification methods to discriminate against various odors because of VOCs. Three pre-handling procedures were used: data difference, data fraction, and data relative. Three sorts of designs were developed in this work: notch design, Bi design, and loading design. These designs ultimately helped differentiate and classify the gases. The loading design was used to decide on the centrality and relationship between MOS sensors. The notch design was used to legitimize the ability of the classifier in separating the smells of each gas. Finally, the Bi design was utilized to picture the interdependency in the middle of perception and reaction. To conclude, the method developed in this study and its ultimate outcomes will enable people working in affected industries to get alerts about the presence of these gases in the environment so that they can take preventative measures.

The proposed system to detect volatile cases was developed on a dataset with a limited number of samples. The number could increase in future studies with more data collection to investigate the proposed method’s generalizability. Future work should study the effect of volatile gases on human health using brain imaging and physiological sensing techniques such as electroencephalogram (EEG) [24,25], electrocardiogram (ECG) [24], heart rate variability analysis (HRV) [26], and magnetic resonance imaging (MRI) [27]. Moreover, another future direction could be to study the discriminating capabilities of machine learning algorithms that classify volatile gases by comparing the performances of a variety of classifiers, such as support vector machines (SVM) [28], gradient boosting (XGB) [29], artificial neural networks (ANN) [30], and k-nearest neighbors (KNN) [31]. Future work on quality assessments of medical herbs like Glycyrrhiza glabra can be done to know the condition of the herb—whether it is in good, over dried, or bad condition.

Using the knowledge gained from the analysis and studies, one can customize the sensor array towards these main volatiles. In this way, one can identify a volatile pattern, which correlates with a better odor discrimination outcome with a lower cost detection risk. This development is very beneficial in biomedical applications in detecting different kinds of diseases because it can correlate key volatiles (potential uremic toxins) to the volatile shift observed in various data records used in medical tests. In this way, one can proceed the approach in this work with applications of low sensors in pathological diagnosis, as it has been found in various literature that the electronic nose is able to distinguish between control blood and “uremic” blood. In addition, the gas sensor series is not only able to discriminate the performance of after-dialysis blood, but it can also follow the unpredictable shift that happens during a single hemodialysis (simply dialysis, a process of purifying the blood of a person whose kidneys are not working normally) session. The e-nose can be used for equally dialysate-side and blood-side monitoring of hemodialysis. In this way, this work has marvelous scope in social welfare and minimizing expenses in costly pathological tests in the diagnosis of diseases.

## Figures and Tables

**Table 1:** Summary of the performance of the classifier for data sensors sets.

Preprocessing Technique	MOS Sensor Set Interaction	MOS Sensor Set Un Interacted	Contrary	Important
Difference	None	{1}, {2}, {3}, {4}, {5}, {6}	{3}, {4}	{3}, {2}, {4}
Fraction	{5,6}	{1}, {2}, {3}, {4}	{3}, {4}, {5}, {6}	{2}, {3}, {4}
Relative	{5,6}	{1}, {2}, {3}, {4}	{1}, {3}, {4}, {5}, {6}	{2}, {3}, {4}

**Table 2:** Comparison with other published work.

Volatile Organic Compounds	Model	Sample/MOS Sensors	Preprocessing Method	References
Ethanol	Exp. Model	25 Samples of beverages	mid infrared spectroscopy with partial least squares regression (MIR-PLS)	[21]
Acetone	Exp. Model	Samples were taken from diabetic patients	Gas Chromatography Mass Spectrometry (GC-MS)	[22]
Propane	Exp. Model	Samples taken from petrochemical storage tanks	PLS	[23]

**Table 3:** Summary of classification results.

Preprocessing Technique	Classifying			Overlapping of Gases
	Acetone + Ethanol	Ethanol + Propane	Acetone + Propane	
Difference	Yes	Yes	Yes	No
Fraction	Yes	Yes	Yes	No
Relative	Yes	Yes	Yes	No

## Appendix A. PCA Variants

(i) **Differential:** The point when the base Y_S_(0) is within sight of drift δA, and then, subtracted from the sensor reaction time Y_S_(t). Furthermore, the present drift δA is smashed. The outline-controlled response Z_S_(t) is given as expressed in the equation below:

Zs(t) = (Ys(t) + δA) − (Ys(0) − δA) = Ys(t) − Ys(0)) (A1)

(ii) **Relative:** By using the relative as one of the methods, a dimensionless response Zs(t) is accomplished, and henceforth, the multiplicative amble δM is abolished. By dividing the sensor response by the baseline, the relative approach can be expressed as:

Zs(t) = Ys(t)(1 + δM)/Ys(0)(1 + δM) = (Ys(t))/(Ys(0)) (A2)

(iii) **Fractional:** When the baseline is subtracted from the response Ys(t) and divided by the baseline Ys(0), a dimensionless and normalized response Zs(t) is achieved from the result of the sensor that goes well for large or small signals. It can be expressed as:

Zs(t) = (Ys(t) − Ys(0))/Ys(0) (A3)

## Appendix B. Literature Review

**Table A1:** Summary of the literature review.

Reference Number	Year	Compounds Analyzed	Technique	Limitations
[6]	2018	Ethanol, propanol, acetone	KMP algorithm	If the extracted features yield poor separability between different classes, the performance of feature extraction is not good.
[7]	2017	Ethanol and acetone	9 different classifiers: K Nearest Neighbors, Linear SVM, radial basis function (RBF) SVM, Decision Tree, Random Forest, AdaBoost, Naive Bayes, linear discriminant analysis (LDA), and quadratic discriminant analysis (QDA).	Results have shown deterioration by up to 30% when the movement speed of the data used for training highly differs from that of the testing.
[11]	2018	Acetone	Gas chromatography- mass spectrometry	Not done on the mixture of gases.
[12]	2007	Ethanol and acetone	Detection device	Cannot separate the mixture of three gases
[14]	2018	Medical field	PCA	Detect chronic diseases and not a mixture of gases.
[15]	2018	Food	Biosensors and electronic tongue	Detect food spoilage.
[18]	2019	CO, CH4, and their mixture	Convolutional neural network	Does not consider the mixture of A, E and P.

## Appendix C. Sensor Array

### C.1. SPECIFICATION OF TGS 2600 SENSOR

The TGS 2600 has a high sensitivity to low concentrations of gaseous air contaminants, such as hydrogen and carbon monoxide, which exist in cigarette smoke. The sensor can detect hydrogen at a level of several ppm. Figaro also offers a microprocessor (FIC02667) that contains special software for handling the sensor’s signal for appliance control applications.

#### C.1.1. Features

The main features of the sensor TGS 2600 are as follows: low power consumption, high sensitivity to gaseous air, contaminants, long life, low cost, use of a simple electrical circuit, and small size.

#### C.1.2. Applications

The sensor TGS 2600 has applications in air cleaners, ventilation control, and air quality monitors.

### C.2. SPECIFICATION OF TGS 2602 SENSOR

The TGS 2602 has a high sensitivity to low concentrations of odorous gases, such as ammonia and H2S generated from waste materials in offices and home environments. The sensor also has a high sensitivity to low concentrations of VOCs, such as toluene emitted from wood finishing and construction products. Figaro also offers a microprocessor (FIC02667) that contains special software for handling the sensor’s signal for appliance control applications.

#### C.2.1. Features

The main features of TGS 2602 are as follows: high sensitivity to VOCs and odorous gases, low power consumption, high sensitivity to gaseous air contaminants, long life, use of a simple electrical circuit, and small size.

#### C.2.2. Applications

The TGS 2602 are used in different applications like air cleaners, ventilation control, air quality monitors, VOC monitors, and odor monitors.

### C.3. SPECIFICATION OF TGS 2611 SENSOR

The TGS 2611 is available in two different models that have different external housings but identical sensitivity to methane gas. Both models can satisfy the requirements of performance standards such as UL1484 and EN50194.

TGS 2611-C00 possesses small size and quick gas response, which makes it suitable for gas leakage checkers. TGS 2611-E00 uses filter material in its housing, which eliminates the influence of interference gases such as alcohol, resulting in a highly selective response to methane gas. This feature makes the sensor ideal for residential gas leakage detectors that require durability and resistance against interference gas.

#### C.3.1. Features

The main features of TGS 2611 are as follows: low power consumption, high sensitivity to methane, long life, low cost, and simple electrical circuit.

#### C.3.2. Applications

The main applications of TGS 2611 are in domestic gas alarms, portable gas detectors, and gas leak detectors for gas appliances.

### C.4. SPECIFICATION OF TGS 2620 SENSOR

The TGS 2620 has a high sensitivity to organic solvent vapors, in addition to other volatile vapors. It also has a sensitivity to several combustible gases (i.e., propane), which makes it a good general-purpose sensor.

#### C.4.1. Features

The main features of TGS 2620 are as follows: low power consumption, high sensitivity to alcohol and organic solvent vapors, long life, low cost, and simple electrical circuit.

#### C.4.2. Applications

The main applications of TGS 2620 are in alcohol testers, organic vapor detectors/alarms, solvent detectors for factories, dry cleaners, and semiconductor industries.

### C.5. SPECIFICATION OF MICS 5135 SENSOR

The use of the MICS 5135 is in VOC measurement applications. The MICS 5135 package targets the detection of reducing gases such as carbon monoxide (CO), hydrocarbons (HC), ethanol, and volatile organic compounds (VOC).

#### Features

The main features of MICS 5135 are low heater current, wide detection range, high sensitivity, fast thermal response, electrostatic discharge protected, miniature dimensions, and high resistance to shocks and vibrations.

### C.6. SPECIFICATION OF MICS 5521 SENSOR

The use of the MICS 5521 is in automotive applications. The package of MICS 5521 also targets the detection of reducing gases like carbon monoxide (CO), hydrocarbons (HC), and volatile organic compounds (VOC). These gases reach significant concentrations in heavy traffic and are responsible for poor cabin air quality.

#### Features

The main features of MICS 5521 are its low heater current, wide detection range, high sensitivity, short pre-heating time, miniature dimensions, and high resistance to shocks and vibrations.
